# Effect of bar designs on peri implant tissues health in implant-supported removable prostheses: a systematic review

**DOI:** 10.1186/s12903-024-03915-5

**Published:** 2024-01-28

**Authors:** Nadine Omeish, Laure Bessou, Maria-Clotilde Carra, Bruno Tavernier, André Luís Porporatti

**Affiliations:** 1 Université Paris Cité and Service of Odontology, Rothschild Hospital, Paris, France; 2 Population-based Epidemiologic Cohorts Unit, Inserm, Villejuif, UMS 011 France; 3 Unité de Recherche Biomatériaux Innovants et Interfaces - URB2i - UR4462, Paris, France; 4 Service of Odontology, Rothschild Hospital, Paris, France; 5 Laboratoire de Neurobiologie Oro-Faciale, Paris, France

**Keywords:** Connecting bar, Dental implants, Peri-implant tissues, Edentulous jaw, Overdenture

## Abstract

Different characteristics of bars (cross-sectional shape, diameter, distal extension etc.) lead to different biomechanical behavior (retention and stress) with implants and peri-implant tissues.

Aim: To evaluate the impact of implant-supported removable prostheses bar designs in fully edentulous arch (in the maxilla and/or mandibula), with 4 implants or more, on the peri-implant soft and hard tissues.

Two reviewers searched for observational studies, RCT and in vitro studies, published on five main databases and three from the grey literature, without restrictions on November 2023.

Of the 3049 selected articles, four met the inclusion criteria. Four RCT evaluated peri-implant health tissues in full edentulous arches with 4 or 6 implants rehabilitated with implant bar overdentures. One prospective study with 5 years follow-up evaluated the success/survival rate of implants and implant bar overdentures. Overall, 261 subjects were enrolled in our systematic review with 1176 implants. Overdentures’ survival rate was 100%. There was a trend that plaque indices and gingival indices were low in all of the studies, however no statistical analysis was done due to the lack of information.

Due to the lack of information in the included studies, we cannot confirm if bar characteristics affect the peri-implant tissues health.

**Supplementary Information:**

The online version contains supplementary material available at 10.1186/s12903-024-03915-5.

## Introduction

According to the McGill consensus, two-implant overdenture (OD) should be the first choice of treatment for the edentulous mandible, regardless of the attachment system used (bars, magnets, balls) [1]. However, in order to enhance treatment outcomes, increasing the number of implants leads to higher retention, less bone loss and better stress distribution [2–5]. As for the maxilla, there is no consensus about the ideal number of implants. Four or six implants have been advocated as the best options to rehabilitate an edentulous maxilla [6, 7]. Implant-retained bar overdentures (IRBOD) with four to six implants without mucosal support, are a well-known treatment for edentulous patients. They represent a valuable option for the rehabilitation of complex situations [8, 9] providing good retention, stability, esthetical asset [6] and good chewing efficiency [10]. Having four implants creates an angular relationship between the implants instead of a straight-line relationship with two implants, which explains the higher retention of the OD [11]. Bars also seem to contribute to load sharing and stress distribution onto the implants [12].

Various bar designs are nowadays available. They differ from their cross-section shape, material, diameter, mucosa-bar distance, and others. These different characteristics of bars lead to different biomechanical behaviors (retention and stress) on implants and peri-implant tissues [13, 14]. Studies show that four-implant bars have a different biomechanical behavior on the cortical bone than two-implant bars [15–18] and that bars, in general, seem to be associated with a higher plaque index and gingival index compared to other attachment systems (ball attachments etc.) [19]. Therefore different bar designs can lead to plaque retention [20].

According to patient-reported outcome measures (PROMs),the ability to maintain oral hygiene is higher in OD than implant-supported fixed prosthesis [21]. Indeed, plaque index, gingival index and probing depth were found to be higher in case of fixed prothesis than OD with 4 implants [22].

Knowing that insufficient plaque control may lead to peri-implant diseases (peri-implantitis and mucositis) [23], the main objective of this systematic review was to answer the following focused question: “What is the impact of implant-supported removable prostheses bar designs in fully edentulous arch (in the maxilla and/or mandibula), with 4 implants or more, on the peri-implant soft and hard tissues?”

## Methods

### Protocol and registration

This systematic review conformed to Preferred Reporting Items for Systematic Reviews and Meta-Analyses PRISMA Checklist [24]. The protocol was registered in the International Prospective Register of Systematic Reviews (PROSPERO) under number CRD42022323998 [25].

### Eligibility criteria

In this review, the inclusion criteria were based on PECOS questions [26]:

Population (P): Adult patients with edentulous maxilla and/or mandibula candidate for implant-supported oral rehabilitation; Exposure (E): 4 or more dental implants with a connecting bar; Comparison (C): Different types of bars (dimensions, gum distance, cross-section shape, number/orientation (tilted or not) of implants, material); Outcome (O): Peri-implant tissues health according to Berglundh &al. 2018 classification [27] or peri-implant mucosa health using Löe and Sillness classification [28] and several indices: plaque index, gingival index, bleeding on probing index, pocket probing depth, calculus index [28, 29], marginal bone loss and implant loss; Study design (S): Experimental (randomized and non-randomized) and observational studies (case-control, cohort, cross-sectional).

No language restrictions were applied on search strategy. Research was performed on November 14th, 2023. The exclusion criteria encompassed: (1) studies with children or animals; (2) studies enrolling patients with a history of oral cancer; (3) studies where dental connecting bars were placed in 3 or less implants; (4) studies with unsplinted implants; (5) studies with fixed prosthodontic bar or with OD with a mucosal support; (6) studies comparing bars with locators or other devices; (7) studies using zirconium, zygomatic or extraoral implants; (8) studies that do not evaluate peri-implant tissues health; (9) studies with no information about the bar design; (10) lack of adequate tool measurements for bone loss (panoramic radiographs) or unknown classifications for indices; 11) clinical studies with a follow-up < 1-year; 12) reviews, letters, systematic reviews, meta-analysis, case reports < 10 subjects, conference abstract, personal opinions; 13) Same studies but different articles.

### Information sources and search strategy

Detailed individual search strategies were developed for each bibliographic electronic database: PubMed (including Medline), Embase, Scopus, and Web of Science. A grey literature search was performed on Google Scholar and Open Grey. All database searches were conducted from the starting coverage date through November 14th, 2023. More information on the search strategies was provided in Appendix 1. Furthermore, the authors hand-searched the reference lists of the selected articles for any additional references that might have been missed in the database searches. All references were managed and the duplicated hits were removed by a reference manager software (EndNote X7® Basic-Thomson Reuters, New York, EUA).

### Selection process

This part followed a two-phase process. In phase-one, two authors (N.O and L.B) independently evaluated the titles and abstracts of all identified citations. In phase-two, the same authors evaluated the selected records on full-text. They independently screened papers on phase-one and -two, applied the eligibility criteria, collected key information from the selected studies, and crosschecked the information. The final selection was based solely on full-text assessment of the studies. When disagreement appeared, a third author (A.L.P) was involved to make a final decision about the inclusion or exclusion of studies.

### Data collection process and data items

For each of the included studies, these data were collected: author(s), year of publication, country, sample size, bar characteristics (diameter, mucosa-bar distance, material, cross-sectional shape, distal extension length), number of implants, type of jaw, results, and main conclusions.

### Study risk of bias assessment

Two methodological appraisal tools were utilized: (1) in observational studies, Joanna Briggs Institute (JBI) critical appraisal checklist was used. It mainly evaluates the confounding bias, the study’s assessment method, and statistical analysis [30]. (2) For Randomized Clinical Trials (RCT), the version 2 of Cochrane Collaboration’s tool for assessing risk of bias was chosen (RoB 2 [31].

Two reviewers (N.O and L.B) scored each item as “yes”, “no” or “unclear”, and classified independently the quality of each included study as “high”, “low” or “unclear” risk of bias. The same two reviewers worked out on any differences regarding data analysis. A third author (A.L.P) was involved to steer decision in case of uncertainty. Figures of the quality assessment of all included studies were generated with Review Manager 5.3 (RevMan 5.3, The Nordic Cochrane Centre, Copenhagen, Denmark).

### Risk of bias across studies and reporting bias assessment

The risk of bias across studies was assessed as an overall risk the study results may present, on which could influence meta-analysis data. Methodological and statistical heterogeneity were evaluated by comparing the variability in study design and the risk of bias.

When the required data were not complete, the reviewer (N.O) attempted to contact the study authors to retrieve any unpublished information. Three attempts were made in a 30 days’ period, by email for the first, second and last author.

### Impact measures and synthesis methods

Any impact on tissues health was evaluated. Mean and standard deviation were used as measure of the impact. If quantitative synthesis was deemed appropriate, a meta-analysis would have been performed by using RevMan 5.3. However, there were not enough data to perform a meta-analysis.

### Certainty assessment

A summary of the overall strength of evidence available was presented using “Grading of Recommendations Assessment, Development and Evaluation” (GRADE) Summary of Findings (SoF) tables, using GRADEpro software [32].

## Results

### Study selection

**Fig. 1:**
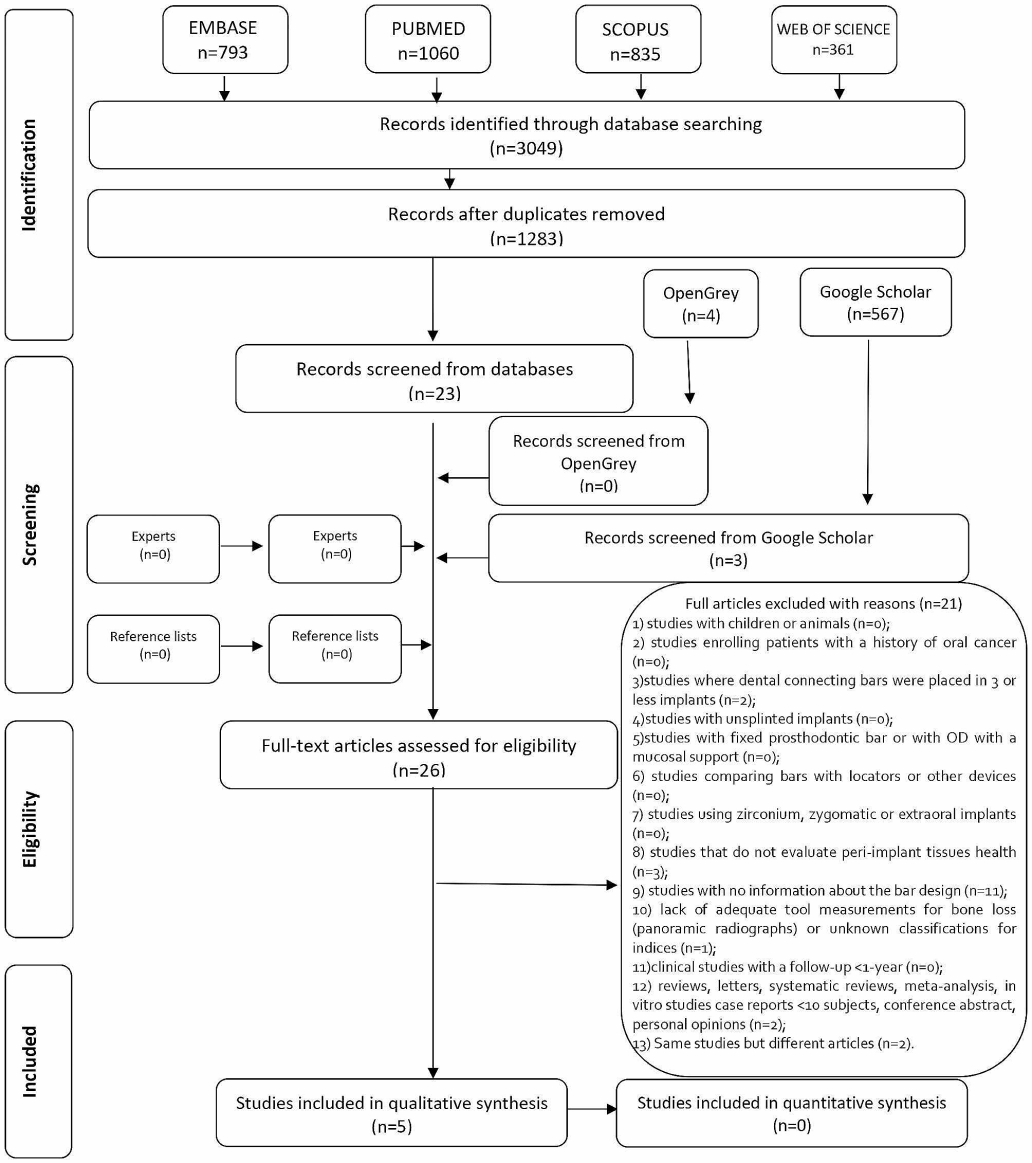
Flow diagram of literature search and selection criteria Adapted from PRISMA

The initial database search identified 3049 studies. After eliminating duplicated hits, 1283 studies remained; 1260 of them were excluded after title and abstract revision, resulting in a final number of 23 articles. Furthermore, 567 studies were found with Google Scholar, and 4 with OpenGrey. Three of them were selected for full-text reading. No additional study was selected from hand-search of the references lists of the included studies. Thus, 26 studies became part of phase-2. During phase-2, a total of 21 studies were excluded (reasons for exclusion may be found in Appendix 2). Five studies were included for qualitative synthesis. A flowchart of the process of identification, inclusion and exclusion of studies is shown in Fig. 1.

### Study characteristics

In total, there was four RCTs [33–36] and one prospective observational study [37]. The mean sample size ranged from 30 [36] to 66 [34] patients with a total of 261 patients receiving a total of 1176 implants. The studies were conducted in Netherland [34, 35], Egypt [33, 36] and in Austria [37].

### Risk of bias assessment

**Fig. 2:**
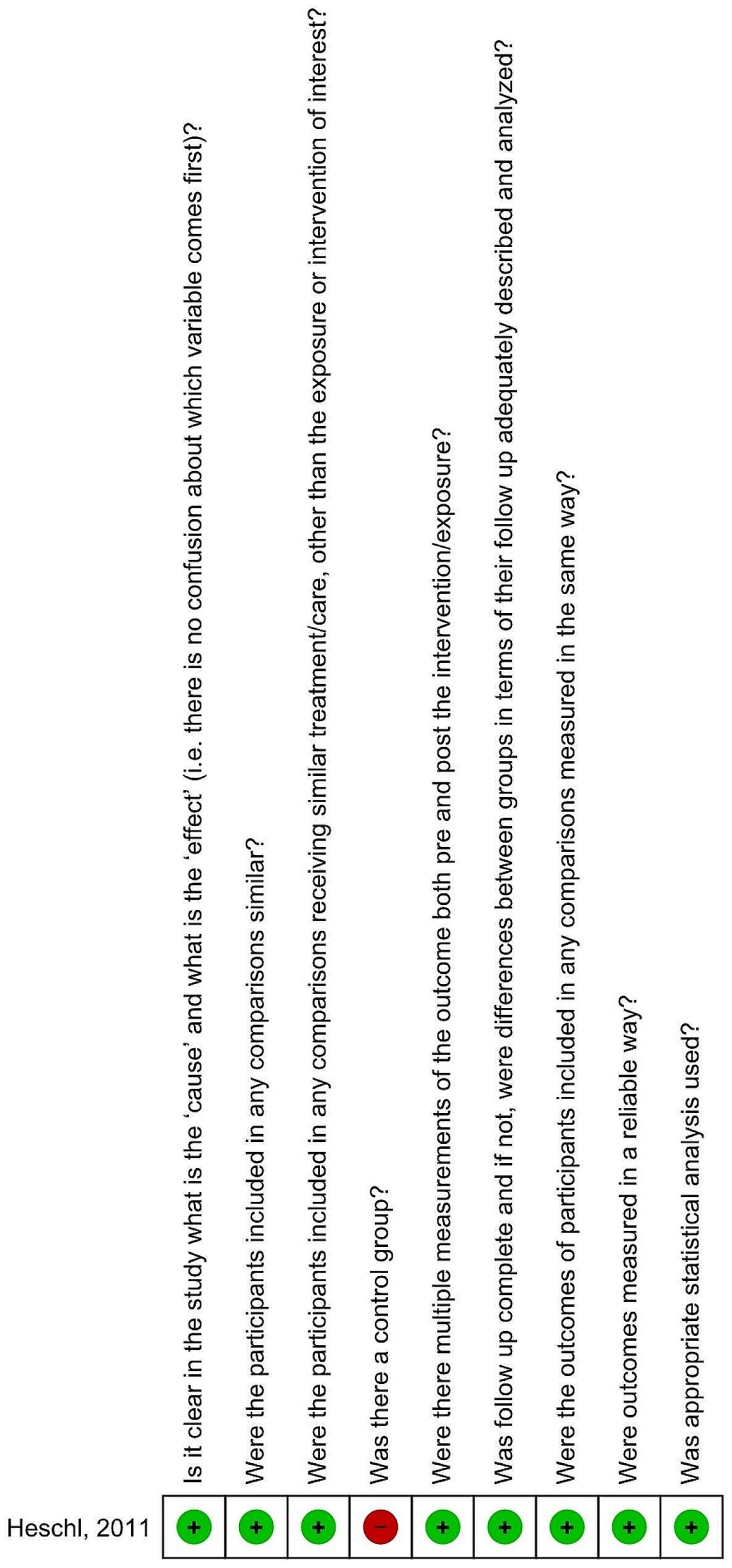
Risk of bias summary of the observational study; Figures generated with Review Manager 5.3 (RevMan 5.3, The Nordic Cochrane Centre, Copenhagen, Denmark, Green plus: Yes, Red minus: No, Yellow question mark: Unclear, White square: not applicable

According to the appropriate tools, the observational study was classified as moderate risk of bias [37], (Fig. 2). Four RCTs were classified as high risk of bias (Table 1, Appendix 3) [33–35].

**Table 1:** Risk of bias for Randomized Clinical Trials. Version 2 of the Cochrane risk-of-bias tool for randomized trials (RoB 2)

	Gibreel, 2017	Slot, 2014	Slot, 2019	Ibrahim, 2022
Was the allocation sequence random?	**Y**	**Y**	**Y**	**Y**
Was the allocation sequence concealed until participants were enrolled and assigned to interventions?	**Y**	**Y**	**Y**	**PY**
Did baseline differences between intervention groups suggest a problem with the randomization process?	**NI**	**PN**	**PN**	**PN**
Risk of bias judgement	**Low Risk**	**Low Risk**	**Low Risk**	**Some concerns**
Were participants aware of their assigned intervention during the trial?	**NA**	**NA**	**NA**	**NA**
Were carers and people delivering the interventions aware of participants’ assigned intervention during the trial?	**NA**	**NA**	**NA**	**NA**
Were there deviations from the intended intervention that arose because of the trial context?	**PY**	**PN**	**PN**	**PN**
Were these deviations likely to have affected the outcome?	**PN**	**PN**	**PN**	**PN**
Were these deviations from intended intervention balanced between groups?	**NI**	**NI**	**NI**	**NI**
Risk of bias judgement	**Some concerns**	**Low Risk**	**Low Risk**	**Low Risk**
Was an appropriate analysis used to estimate the effect of assignment to intervention?	**Y**	**Y**	**Y**	**Y**
Was there potential for a substantial impact (on the result) of the failure to analyse participants in the group to which they were randomized?	**NA**	**NA**	**NA**	**NA**
Risk of bias judgement	**Low Risk**	**Low Risk**	**Low Risk**	**Low Risk**
Was an appropriate analysis used to estimate the effect of adhering to intervention?	**NI**	**NI**	**NI**	**Y**
Risk of bias judgement	**Some concerns**	**Some concerns**	**Some concerns**	**Low Risk**
Were data for this outcome available for all, or nearly all, participants randomized?	**NI**	**NI**	**NI**	**NI**
Is there evidence that the result was not biased by missing outcome data?	**N**	**N**	**N**	**N**
Could missingness in the outcome depend on its true value?	**NI**	**NI**	**NI**	**NI**
Is it likely that missingness in the outcome depended on its true value?	**NI**	**NI**	**NI**	**NI**
Risk of bias judgement	**High Risk**	**High Risk**	**High Risk**	**High Risk**
Was the method of measuring the outcome inappropriate?	**N**	**N**	**N**	**N**
Could measurement or ascertainment of the outcome have differed between intervention groups?	**N**	**N**	**N**	**N**
Were outcome assessors aware of the intervention received by study participants?	**NA**	**NA**	**NA**	**NA**
Could assessment of the outcome have been influenced by knowledge of intervention received?	**PN**	**PN**	**PN**	**PN**
Is it likely that assessment of the outcome was influenced by knowledge of intervention received?	**PN**	**PN**	**PN**	**PN**
Risk of bias judgement	**Low Risk**	**Low Risk**	**Low Risk**	**Low Risk**
Were the data that produced this result analysed in accordance with a pre-specified analysis plan that was finalized before unblinded outcome data were available for analysis?	**NI**	**NI**	**NI**	**NI**
Is the numerical result being assessed likely to have been selected, on the basis of the results, from multiple eligible outcome measurements within the outcome domain?	**PN**	**PN**	**PN**	**PN**
Is the numerical result being assessed likely to have been selected, on the basis of the results, from multiple eligible analyses of the data?	**PN**	**PN**	**PN**	**PN**
Risk of bias judgement	**Some concerns**	**Some concerns**	**Some concerns**	**Some concerns**
**Overall risk of bias judgement**	**High Risk**	**High Risk**	**High Risk**	**High Risk**

Legend: N = No, PN = Probably No, Y = Yes, PY = Probably Yes, NI = No Information, NA = Not Applicable

### Descriptive synthesis of the included studies

Overall, 261 subjects were enrolled in our systematic review with 1176 implants (approximately 5 implants per patient). The follow-up period were 1 to 5 years. Six implants were lost in total. According to thre of the clinical studies [34, 35, 37], OD survival rate was 100%. There was a trend that plaque indices and gingival indices were low (Table 2) in all of the studies, however no statistical analysis was done due to the lack of data.

Outcomes and main results of each study are reported in Table 2A (for observational studies), Table 2B (for RCT). Among the included studies, none assessed the impact of bar designs on peri-implant tissue health as a main objective.

**Table 2A:** Summary of descriptive characteristics of included Observational Studies (*n* = 1)

Author, year	Type of study	Number of patients	Groups	Clinical parameters	Outcomes	Results
Heschl & al., 2011	Retrospective study (5 years follow-up)	39 patients with 4 implants each	Only one group, each subject with 4 implants	-Arch: mandible; -Distal extension: max 12 mm; -Material: no information; -Cross-sectional shape : Dolder bar; - Width: no information - Distance bar/mucosa : no information -Use of abutments : yes -Other informations: splinted bar, gold copings	-Marginal bone loss (long cone paralleling technique), - Plaque Index Mombelli 1987 -Bleeding Index Mombelli 1987 -Probing pocket depth -Peri implantitis and mucositis Löe & Silness 1963	-Mean of bone loss = 1.44(0.78) -Plaque Index = 0.5(0.5) -Calculus Index = 0.5(0.3) -Gingival Index = 0.7(0.4) -Bleeding Index = 1.3(0.6) -Probing depth in mm = 3.8(0.6) -Implant fails = 1 -Implant losses = 1

**Table 2B:** Summary of descriptive characteristics of included Randomized Clinical Trial Studies (*n* = 4)

Author, year	Type of study	Total of inclusions	Groups	Clinical parameters	Outcomes	Results
Slot & al. 2014	RCT (1 year follow-up)	*N* = 66	Group A with 4 implants (*n* = 33) vs. group B with 6 implants (*n* = 33)	-Arch: maxilla and mandible for each patient; -Distal extension: no but mesial extension : 8 mm; -Material: titanium; - Cross-sectional shape: egg-shaped bar; -Width: 2.19 mm; -Distance bar/mucosa: 2 mm - Use of abutments: yes -Other informations: milled bar; two bars; splinted bar but unsplinted between the medial implants;	-Peri-implant bone level (long cone paralleling technique) -Plaque Index by Mombelli 1987 -Calculus index (0 or 1) -Peri-implant inflammation by Löe and Sillness 1963 -Bleeding Index: Mombelli 1987 -Probing Pocket Depths: 4 sites	-Mean of bone loss in group A: 0.35 (SD: 0.31) -Mean of bone loss in group B: 0.46 (SD: 0.34) -Plaque Index: group A = 0; group B = 0 -Calculus Index: group A = 0; group B = 0 -Gingival Index: group A = 0; group B = 0 -Bleeding Index: group A = 0; group B = 1 -Probing depth in mm: group A = 4.8; group B = 4.4 -Implant losses in group A = 0 -Implant losses in group B = 1
Gibreel & al. 2017	RCT (1 year follow-up)	*N* = 60	-Group A : with clips (*n* = 30) vs. group B : resilient liner (*n* = 30)	-Arch: maxilla -Distal extension: no; -Material: titanium; - Cross-sectional shape: round -Width: no information; -Distance bar/mucosa: no information; -Use of abutments : yes Other informations: two bars in the same jaw; gold clips or resilient soft liner; instant adjusted bar	- Modified Plaque Index by Mombelli 1987 -Modified Bleeding Index: Mombelli 1987 -Probing Pocket Depths: 4 sites	-Modified Plaque Index, *- > anterior implants*: group A = 0.88(0.043); group B = 0.12(0.01) *-> Posterior implants*: group A = 0.97(0.063); group B = 0.12(0.007) -Modified Bleeding Index, *- > anterior implants*: group A = 0.22(0.01); group B = 0.015(0.02) *-> Posterior implants*: group A = 0.27(0.01); group B = 0.019(0.03) -Probing Pocket Depths, *- > anterior implants*: group A = 1.91(0.073); group B = 0.63(0.056) *-> Posterior implants*: group A = 2.13(0.073); group B = 0.82(0.078) -2 implant failures in GI.
Slot & al. 2019	RCT (1 year follow-up)	*N* = 66	Group A with 4 implants (*n* = 33) vs. group B with 6 implants (*n* = 33)	-Arch: maxilla and mandible for each patient; -Distal extension: no but mesial extension : 8 mm; -Material: titanium; - Cross-section shape: egg-shaped bar; -Width: 2.19 mm; -Distance bar/mucosa: 2 mm - Use of abutments: yes -Other informations: milled bar; two bars; splinted bar but unsplinted between the medial implants;	-Peri-implant bone level (long cone paralleling technique) -Plaque Index by Mombelli 1987 -Calculus index (0 or 1) -Peri-implant inflammation by Löe and Sillness 1963 -Bleeding Index: Mombelli 1987 -Probing Pocket Depths: 4 sites	-Mean of bone loss ->group A: 0.58(SD: 0.51) ->group B: 0.60(SD: 0.58) -Plaque Index: -> group A, 0 = 62.1%, 1 = 20.7%, 2 = 17.2%, 3 = 0 ->group B, 0 = 51.6%, 1 = 25.8%, 2 = 22.6%, 3 = 0 -Calculus Index: -> group A, 0 = 96.6%, 1 = 3.4% ->group B, 0 = 96.8%, 1 = 3.2% -Gingival index: -> group A, 0 = 48.3%, 1 = 41.4%, 2 = 10.3%, 3 = 0 ->group B, 0 = 54.8%, 1 = 41.9% 2 = 3.3%, 3 = 0 -Bleeding Index: -> group A, 0 = 51.7%, 1 = 37.9%, 2 = 10.4%, 3 = 0 ->group B, 0 = 38.7%, 1 = 41.9%, 2 = 19.4%, 3 = 0 -Probing depth in mm: group A = 4.3(1.0); group B = 4.2(0.8) -Implant losses in group A = 0 -Implant losses in group B = 1
Ibrahim et al. 2022	RCT (1 year follow-up	*N* = 30	Group A with 4 vertical implants (*n* = 15) Group B with 2 vertical anterior implants and 2 posterior implants tilted 30° distally (*n* = 15)	-Arch: mandible; -Distal extension: 7 mm; -Material: no information; - Cross-sectional shape: Hader bar; -Width: no information; -Distance bar/mucosa: 2 mm - Use of abutments: yes -Other informations: resin clips	-Peri-implant bone level (CBCT) -Plaque Index by Mombelli 1987 -Gingival Index by Mombelli 1987 -Probing Pocket Depths: 4 sites	*For the anterior implants* -Peri-implant bone level: *For the anterior implants* -> group A = 0.50 mm -> group B = 0.47 mm *For the posterior implants* *->* group A = 0.90 mm -> group B = 0.45 mm -Plaque Index: *For the anterior implants* -> group A = 3 -> group B = 1 *For the posterior implants* *->* group A = 1 -> group B = 0 -Gingival Index: *For the anterior implants* -> group A = 0 -> group B = 0 *For the posterior implants* *->* group A = 0 -> group B = 1 -Probing Pocket Depths: *For the anterior implants* -> group A = 2.30 mm -> group B = 1.80 mm *For the posterior implants* *->* group A = 1.85 mm -> group B = 1.56 mm

Heschl &al. 2013 [37], conducted a prospective study with a 5 years follow-up. Their main objective was to evaluate the outcomes of 4 or 6 Xive® S plus implants (Dentsply Friadent, Mannheim, Germany) following conventional restoration with IRBOD in the mandible. After 5 years, the implants success rate was 98.4% and the prosthesis success rate was 75% but the survival rate was 100%. Mean scores of pocket probing depth, indices for plaque, calculus, gingiva, and bleeding were very low after 1-year of loading, and did not significantly differ throughout the 5 years follow-up.

Slot et al. 2014 [34], compared the treatment outcome of 4 implants vs. 6 in the posterior region of the maxilla with IRBOD after a 1 year functional period. Survival rate of 4 implants was 100% vs. 99.5% for 6 implants. OD survival rate was 100%. There was no significant difference between both groups concerning the periodontal indices, marginal bone loss and probing depth.

As for Slot et al. 2019 [35], they compared 4 vs. 6 implants OD in the maxilla after a 5 year observation period. We noted that there is a high chance that it might be the same study as 2014 [34]. The main results are the same except for the 6 implants survival rate which was 99.2%. There was no significant difference between both groups concerning the periodontal indices, marginal bone loss and probing depth.

Gibreel et al. 2017 [33], compared bar-clips vs. silicone-resilient liners used with IRBOD with 4 implants in the mandible after a one year follow-up period. Two implant failures were noted in the bar-clip group vs. 0 in the resilient liner group. Plaque index, bleeding index and probing pocket depths were significantly higher in the bar-clip group.

Ibrahim et al. 2022 [36], compared four vertical implants versus two anterior vertical implants/two posterior implants tilted 30° distally, after a one year follow-up period. Overall, implants survival rate was 100%. The vertical implants group had significantly higher peri-implant bone loss and higher pocket depths than the tilted implants group. Plaque index (PI) and gingival index (GI) were significantly higher in the vertical group except in the posterior implants it was significantly lower in the vertical group. Two dentures were fractured in the tilted implants group.

### Results of syntheses

A meta-analysis was not performed because of the insufficiency of data for statistical pooling related to the sort of study included and the heterogeneity of the studies included.

### Certainty of evidence

The overall quality of evidence identified using GRADE’s SoF tables was assessed as very low (Appendix 4), because of lack of control group for observational studies; and there were no information about the dropouts and not enough data about the statistical analysis for RCT. Furthermore, study design labeled as observational studies consistently downgraded the certainty.

## Discussion

Within the limitation of the current literature, and based on the included studies, there is insufficient evidence to determine whether bars characteristics impact the peri-implant tissues health in case of a four or six implant-bar.

However, studies have been led in case of two-implant bars and the effect of their designs on peri-implant tissues (mucosa and bone). Stoker et al. 2012 [38] suggested in their study (comparing two versus four-implant bars) that pocket depths could be explained by gingival hyperplasia due to the bar design around the abutments and the mucosa-bar distance. In Phillips et al. 2001, the higher the mucosa-bar distance the better the hygiene is around implants. A 1 mm distance is required for good plaque control [20]. However, in vitro, the higher mucosa-bar distance the higher the stress is on the peri-implant bone [39, 40]. One to 2 mm bar height seems to exert an acceptable stress distribution around the peri-implant bone and to avoid any peri-implant tissue inflammation [20, 41]. In three of the included studies the mucosa-bar distance was 2 mm [34–36]. No data was mentioned for the other studies.

In case of two-implant bars, the higher the distal extension length, the more stress it causes on the peri-implant bone [42–44]. One clinical study recommends a 7 mm distal extension in order to prevent a high strain around the two implants [43]. In this systematic review, one study had a 12 mm distal extension [37], two other studies an 8 mm mesial extension (using two bars) [34, 35]. One of the included studies [36] compared a 7 mm extension (with 30° tilted implants) versus no extension (vertical implants). The implants success rate was 100% but the PI and GI were significantly higher in the no extension group. This could be due to the higher inter-implant distance in case of tilted implants. Francetti et al.2008 [45] suggest that the lower the distance between implants the easier it is for the patient to clean and the lower the PI.

To our knowledge, no clinical study was led in case of four-implant bar with different distal extension lengths. Studies seem to differ if distal extensions (with four implants) are related to high gingival index and plaque index score and marginal bone loss. Some studies conclude that bars with distal extensions seem to enhance peri-implantitis [45]. On the other hand, Krenmair et al. 2007 [47] showed that distal extensions (10 mm) did not affect distal bone loss nor implant survival rate in milled bars. These results are in line with Ibrahim et al.2022 study mentioned previously.

According to Al Qutaibi et al. 2020 [48] meta-analysis comparing marginal bone loss in two versus four-implants bars overdentures, no significant difference was detected in the marginal bone loss of the selected studies [5, 38, 49, 50] between both groups. However, three [5, 49, 50] of the included articles had the same study using a round shaped bar with no distal extension and a mucosa-bar distance higher than 2 mm. One of these studies [5] had a one year follow-up period and reported no implant failure in case of four-implants bars. One of the included studies in this review [33] used bars with the same characteristics (round shaped bars, no distal extension, clips) and did not report any implant failure after a one-year follow-up either.

Abdel Dayem et al. 2009 RCT [51] compared two implants prefabricated round bars OD and custom-made bars. There seem to be less bone resorption in prefabricated round bars with clips and lower gingival index and plaque index score. However, no implants were lost after an 18 months follow-up period. In Gibreel et al. 2017 [33], one of the included RCT in this review, used the same prefabricated bar. Two implants were lost after a one year follow-up in the “bar-clip” group. Authors suggest that these results may be attributed to plaque accumulation due to the space under the bar and around the abutments, leading to gingival inflammation and to difficulties for the patients to maintain adequate oral hygiene around abutments.

In one of the excluded studies (due to the lack of adequate tool measurements for bone loss), authors retrospectively compared round prefabricated bars vs. single anterior milled bars vs. two bilaterally placed milled bars on four to six implants. Peri-implantitis were found in nine implants in the second group (anterior milled bar) vs. one and two in the first and third group. According to the authors, the bar designs might explain the high peri-implantitis rate since it might lead to a limited access for oral hygiene [46]. However, 37% of the participants were smokers which also could explain the high peri-implantitis rate.

It is now well-known that implants overload may lead to marginal bone loss [52, 53]. Various in vitro studies evaluated the impact of different bars configuration on the peri-implant bone. The cross-section bar shape seems to influence the stress around implants: the stress transferred in the bone for a rectangular profile is higher than a round, an L-shape and a square profile. Square profile exerted the less stress [14]. Two of the included studies used egg-shaped bars [34, 35], one study used Hader bars [36], another study used round prefabricated bars [33] and one study used Dolder bars [37]. The higher the diameter of the bar the less stress is exerted on the peri-implant bone but there is no statistical difference between 4 and 6 mm [14]. In the included studies of this review, only two studies mention the bars diameters (2.19 mm for both) [34, 35].

In de la Rosa’s in vitro study [14], the effect of overloaded forces on implants (235 N) was evaluated. The stress on the peri-implant bone varied between 9.9 MPa (Co-Cr round bar, 4 mm diameter, 4 vertical implants, no distal extension) and 79.5 MPa (Titanium grade 5 round bar, 0.5 mm diameter, 4 vertical implants, no distal extension). According to Bozkaya &al. 2004 [54], the ultimate stress of the cortical bone is 100 MPa in tension and 170 MPa in compression. None of these values were reached in de la Rosa’s et al. 2019 study. Thus even if bar characteristics tend to have an impact on the stress of the peri-implant bone, the stress values may not exceed the overloading conditions of the cortical bone.

A study showed that the stiffer the framework bar material, the higher the stress is on the peri-implant bone [55]. Titanium bars or Co-Cr bars have no influence on the peri-implant bone stress [52]. In de la Rosa’s et al. 2019 study, there was a difference in the bone stress around four implants in case of a Ti bar and a Co-Cr bar, but again the amount of stress did not exceed the overloading conditions on the cortical bone. We did not have enough data concerning the framework material of the bars (only two studies reported using titanium bars [34, 35]).

Two bars/4 implants exert higher stress on the bone than 1 bar/4 implants [56]. Three of the included studies [33–35] used 2 bars/4 implants OD and two studies used 1 bar/4 implants OD [36, 37]. But the implant survival rate was high (97–100%) and the plaque indices low (from 0 to 1 according to Mombelli’s classification [29]).

There are many other factors that can influence the peri-implant tissues health, besides bars characteristics such as: excessive retention of the OD [13], the use of abutments, type of clips, implants orientation etc. An in vitro study [52], showed that the use of multiunit or converting adapters reduces the stress around the peri implant bone, in case of 2 implants bar. In this systematic review, all of the studies used abutments [33–35, 37]. Properties of the abutment materials seem to influence the peri-implant bone stress [57]. Composite materials seem to be the most shock-absorbing compared to titanium [57, 58]. We lack information about the abutment’s material in the included studies.

The higher the vertical misfit the higher the stress around implants [59]. All of the included studies did not evaluate any vertical misfit of the bar around the abutments/bar.

Tilted implants (45°) exert higher stress on the bone than vertical implants, in case of 4 implants [14, 17, 60]. Only one study [36] had a group with tilted implants (30°). Compared to the vertical implants group, peri-implant bone loss was significantly lower. Authors explain this by the fact that the increase of anterior-posterior spread provides a wider load distribution and thus less peri-implant bone stress.

Clips material also influences the stress around implants. In a two implants in vitro study [59], plastic clips exerted less stress than gold clips on the peri-implant bone. Four studies used gold clips [33–35, 37] and one study used resin clips [36].

Another factor that influences the stress around the peri-implant bone, is the antagonistic arch [57]. Natural teeth absorb better the shock of the mastication than implant prosthesis [61]. Two studies included patients with full edentulism in the antagonist arch and had implant-OD with 4 implants [34, 35] and two studies included patients with an antagonist arch using a conventional complete denture [33, 36].

## Conclusions

Due to the lack of information in the included studies, we cannot confirm if bar characteristics affect the peri-implant tissues health. Overall, plaque indices and gingival indices, also implant losses, did not seem to differ between the clinical studies selected in the present systematic review. More RCTs and observational studies are required to study directly the effect of bars characteristics on peri-implant tissues health in full edentulous arch with four or more implants.

### Electronic supplementary material

Below is the link to the electronic supplementary material.

**Supplementary Material 1: Appendix 1:** Database search strategy (November 14th, 2023)

**Supplementary Material 2: Appendix 2:** Articles excluded and the reasons for exclusion (n=21)

**Supplementary Material 3: Appendix 3:** Cochrane Collaboration’s tool for assessing risk of bias (RoB 2). (A) Risk of bias summary; (B) Risk of bias graph

**Supplementary Material 4: Appendix 4:** Summary of the overall strength of evidence using Grading of Recommendations Assessment, Development and Evaluation (GRADE)

## Data Availability

The datasets used and/or analysed during the current study available from the corresponding author on reasonable request.
