# Supplementary material for: Effect of bar designs on peri implant tissues health in implant-supported removable prostheses: a systematic review
Source: BMC Oral Health. 2024 Jan 28;24:138. doi: 10.1186/s12903-024-03915-5 (PMC10822188; doi:10.1186/s12903-024-03915-5)
Supplement: Supplementary file 1 — Supplementary Material 1: Appendix 1: Database search strategy (November 14th, 2023) [file 12903_2024_3915_MOESM1_ESM.docx]

**Appendix 1** - Database search strategy (February 7^rd^, 2022)

| **Database** | **Search** |
| --- | --- |
| **PubMed** | ("Connecting bar" OR bar OR bars) AND (prosthesis[MeSH Terms] OR prosthesis OR "dental prosthesis"[MeSH Terms] OR "dental prosthesis" OR denture[MeSH Terms] OR denture OR dentures OR overdenture OR overdentures OR "Dental Prosthesis, Implant-Supported"[MeSH Terms] OR "Dental Prosthesis, Implant-Supported" OR "denture, overlay"[MeSH Terms] OR "denture, overlay") AND ("dental implants" OR "dental implants"[MeSH Terms] OR "dental implantation"[MeSH Terms] OR "dental implantation" OR "dental implantations") |
| **Scopus** | ("Connecting bar" OR bar OR bars) AND prosthesis OR "dental prosthesis" OR denture OR dentures OR overdenture OR overdentures OR "Dental Prosthesis, Implant-Supported" OR "denture, overlay") AND ("dental implants" OR "dental implantation" OR "dental implantations") |
| **Web of Science** | ("Connecting bar" OR bar OR bars) AND (prosthesis OR "dental prosthesis" OR denture OR dentures OR overdenture OR overdentures OR "Dental Prosthesis, Implant-Supported" OR "denture, overlay") AND ("dental implants" OR "dental implantation" OR "dental implantations") |
| **Embase** | ("Connecting bar" OR bar OR bars) AND (prosthesis OR "dental prosthesis" OR denture OR dentures OR overdenture OR overdentures OR "Dental Prosthesis, Implant-Supported" OR "denture, overlay") AND ("dental implants" OR "dental implantation" OR "dental implantations") |
| **Google Scholar** | "connecting bar" AND implant AND health |
| **OpenGrey** | connecting bar |
