# Supplementary material for: Effect of bar designs on peri implant tissues health in implant-supported removable prostheses: a systematic review
Source: BMC Oral Health. 2024 Jan 28;24:138. doi: 10.1186/s12903-024-03915-5 (PMC10822188; doi:10.1186/s12903-024-03915-5)
Supplement: Supplementary file 2 — Supplementary Material 2: Appendix 2: Articles excluded and the reasons for exclusion (n=21) [file 12903_2024_3915_MOESM2_ESM.docx]

**Appendix 4:** Summary of the overall strength of evidence using Grading of Recommendations Assessment, Development and Evaluation (GRADE).

| **Certainty assessment** | | | | | | | **Certainty** | **Importance** |
| --- | --- | --- | --- | --- | --- | --- | --- | --- |
| **№ of studies** | **Study design** | **Risk of bias** | **Inconsistency** | **Indirectness** | **Imprecision** | **Other considerations** |  |  |
| **Observational studies** | | | | | | |  |  |
| 1 | observational studies | serious^a^ | not serious | serious | not serious | none | ⨁◯◯◯ Very low | IMPORTANT |
| **Randomised Clinical Trials** | | | | | | |  |  |
| 4 | randomised trials | very serious^c^ | not serious | serious^d^ | not serious | none | ⨁◯◯◯ Very low | IMPORTANT |

**CI:** confidence interval

#### Explanations

a. The study had not presented a control group

b. The risk of bias were high for some questions (95% CI, trial limitations) and no statistical analysis was done in one of the studies.

c. In all 3 RCT, no information about the dropouts and not enough data about the statistical analysis done concerning the dropouts.

d. The main objectives of the RCT are different from the main objective of this review.
