# Supplementary material for: Effect of bar designs on peri implant tissues health in implant-supported removable prostheses: a systematic review
Source: BMC Oral Health. 2024 Jan 28;24:138. doi: 10.1186/s12903-024-03915-5 (PMC10822188; doi:10.1186/s12903-024-03915-5)
Supplement: Supplementary file 3 — Supplementary Material 3: Appendix 3: Cochrane Collaboration’s tool for assessing risk of bias (RoB 2). (A) Risk of bias summary; (B) Risk of bias graph [file 12903_2024_3915_MOESM3_ESM.docx]

**Appendix 2 -** Articles excluded and the reasons for exclusion (n=21).

| **Author, year** | **Reasons for exclusion*** |
| --- | --- |
| 1. Abraboh, 2020 | 9 |
| 1. Bidez, 1993 | 8 |
| 1. De Medeiros, 2017 | 9 |
| 1. Elsyad, 2017 | 8 |
| 1. Jagger, 2001 | 9 |
| 1. Kim, 2016 | 9 |
| 1. Kumar, 2013 | 3 |
| 1. Lehmann, 2013 | 9 |
| 1. Naert, 1998 | 9 |
| 1. Pan, 2013 | 9 |
| 1. Slot, 2012 | 13 |
| 1. Slot, 2013 | 9 |
| 1. Slot, 2016 | 13 |
| 1. Tanoue, 2016 | 8 |
| 1. Williams, 2001 | 9 |
| 1. Meriscke-Stern, 1994 | 9 |
| 1. Oetterli, 2001 | 3 |
| 1. Eckert, 2001 | 9 |
| 1. Rinke, 2015 | 10 |
| 1. De la Rosa Castotolo, 2019 | 12 |
| 1. Mochalski, 2021 | 12 |

1) studies with children or animals; 2) studies enrolling patients with a history of oral cancer; 3) studies where dental connecting bars were placed in 3 or less implants; 4) studies with unsplinted implants; 5) studies with fixed prosthodontic bar or with OD with a mucosal support; 6) studies comparing bars with locators or other devices; 7) studies using zirconium, zygomatic or extraoral implants; 8) studies that do not evaluate peri-implant tissues health; 9) studies with no information about the bar design;10) lack of adequate tool measurements for bone loss (panoramic radiographs) or unknown classifications for indices; 11) clinical studies with a follow-up <1-year; 12) reviews, letters, systematic reviews, meta-analysis, in vitro studies, case reports <10 subjects, conference abstract, personal opinions; 13) Same studies but different articles.
