# Supplementary material for: Effect of bar designs on peri implant tissues health in implant-supported removable prostheses: a systematic review
Source: BMC Oral Health. 2024 Jan 28;24:138. doi: 10.1186/s12903-024-03915-5 (PMC10822188; doi:10.1186/s12903-024-03915-5)

**Appendix 3 Figure S1 -** Cochrane Collaboration’s tool for assessing risk of bias (RoB 2). (A) Risk of bias summary; (B) Risk of bias graph.

**(A)**
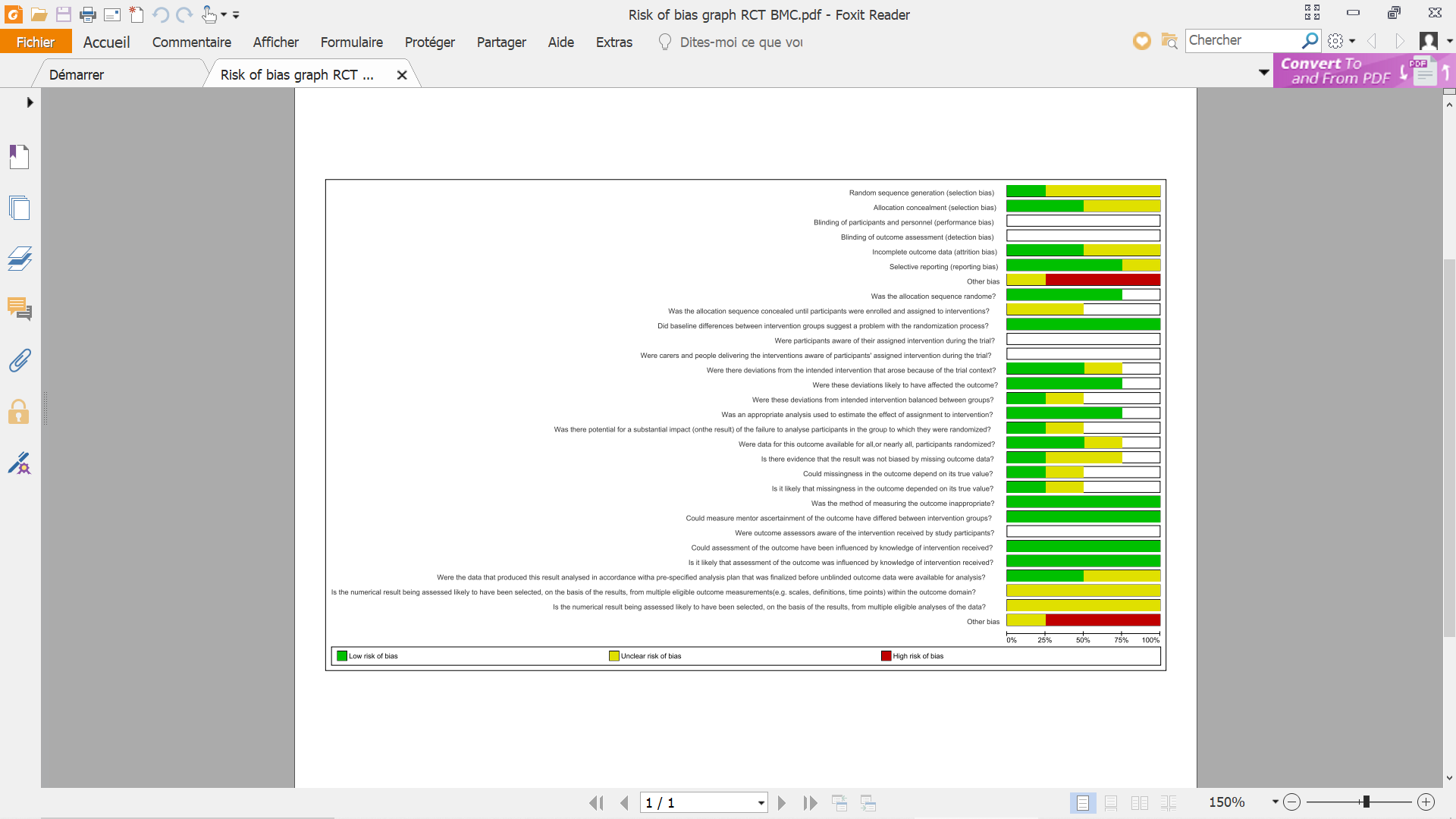


**B)**
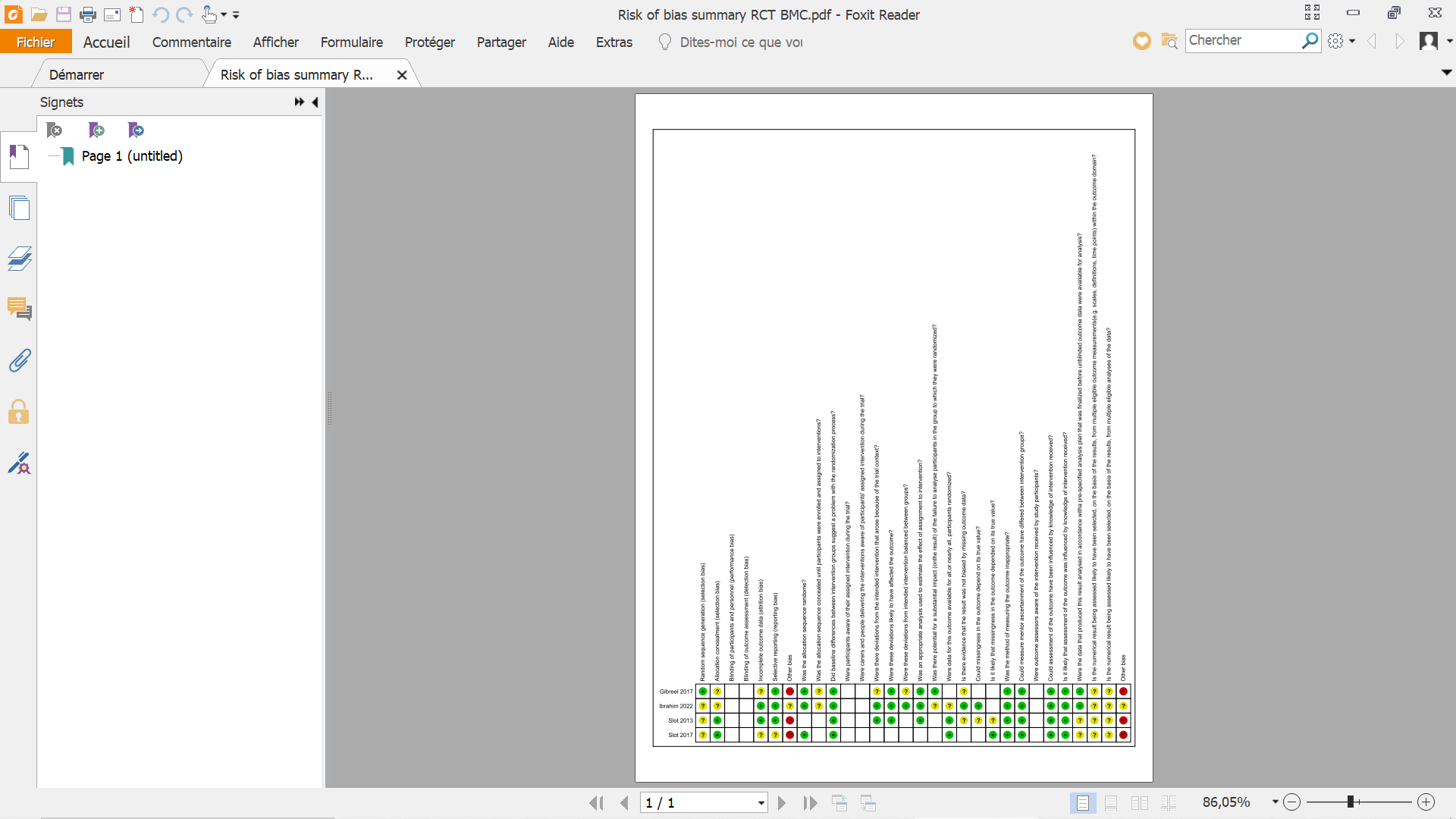

Supplement: Supplementary file 4 — Supplementary Material 4: Appendix 4: Summary of the overall strength of evidence using Grading of Recommendations Assessment, Development and Evaluation (GRADE) [file 12903_2024_3915_MOESM4_ESM.docx]
